# Honey bees (*Apis mellifera spp*.*)* respond to increased aluminum exposure in their foraging choice, motility, and circadian rhythmicity

**DOI:** 10.1371/journal.pone.0218365

**Published:** 2019-06-27

**Authors:** Ana M. Chicas-Mosier, Christopher W. Dinges, Jose L. Agosto-Rivera, Tugrul Giray, Devrim Oskay, Charles I. Abramson

**Affiliations:** 1 Oklahoma State University Department of Integrative Biology, Stillwater, Oklahoma, United States of America; 2 Oklahoma State University Department of Psychology, Stillwater, Oklahoma, United States of America; 3 University of Puerto Rico at Rio Piedras Department of Biology, San Juan, Puerto Rico, United States of America; 4 Namık Kemal Üniversitesi Değirmenaltı Campus Department of Agricultural Biotechnology, Tekirdağ, Turkey; Universitat Leipzig, GERMANY

## Abstract

Aluminum is increasingly globally bioavailable with acidification from industrial emissions and poor mining practices. This bioavailability increases uptake by flora, contaminating products such as fruit, pollen, and nectar. Concentrations of aluminum in fruit and pollen have been reported between 0.05 and 670mg/L in North America. This is particularly concerning for pollinators that ingest pollen and nectar. Honey bees represent a globally present species experiencing decline in Europe and North America. Region specific decline may be a result of differential toxicity of exposure between subspecies. We find that European honey bees (*Apis mellifera mellifera*) may have differential toxicity as compared to two allopatric Mediterranean subspecies (*Apis mellifera carnica* and *Apis mellifera caucasica*) which showed no within subspecies exposure differences. European honey bees were then used in a laboratory experiment and exposed to aluminum in their daily water supply to mimic nectar contamination at several concentrations. After approximately 3 weeks of aluminum ingestion these bees showed significantly shorter captive longevity than controls at concentrations as low as 10.4mg/L and showed a possible hormetic response in motility. We also compared European honey bees to Africanized/European hybrid bees (*Apis mellifera mellifera/scutellata* hybrid) in short-term free-flight experiments. Neither the European honey bee nor the hybrid showed immediate foraging deficits in flight time, color choice, or floral manipulation after aluminum exposure. We conclude that European honey bees are at the greatest risk of aluminum related decline from chronic ingestion as compared to other subspecies and offer new methods for future use in honey bee toxicology.

## Introduction

Pollinator stress is attributed to three primary factors: 1) habitat fragmentation, 2) chemical application and 3) introduced pathogens [1–4]. These factors can be cooperative and can create unmanageable stress and reduce population survival. For example, insecticide application may decrease immunity, allowing pathogens to more easily incapacitate pollinators [5, 6]. Stress caused by pathogens may then be exacerbated by poor foraging from contaminant exposure or environmental factors. Inefficient foraging is a documented result of neonicotinoid insecticide exposure through reduced olfactory senses and the cholinergic system [7–10]. As a result of the stress placed on pollinators, some neonicotinoids have been banned in Europe [11, 12]. Unfortunately however, virtually identical mechanisms of action and consequences of other toxicant exposure, such as from metals, are not as well studied and may have similar negative impacts on pollinators. This article seeks to determine how aluminum affects the choice-making, motility, circadian rhythmicity and lifespan of honey bee subspecies in free-flight and captive laboratory conditions.

### Aluminum rationale

Research suggests that metal ingestion can be a risk to pollinators. Metal exposure can occur through mishandling of waste-water, mining residuals, or acidification [13–15]. Plants can then take up metals through the same mechanisms as micronutrient intake; plants then distribute the compounds through pollen and nectar [14, 16]. Exposure to lead and cadmium through these means have been shown to cause increased metallothionein production and α-tocopherol in pollinator species [13]. In addition to known metal exposure pathways, honey bees have been indicated as potential bio-indicators for metal exposure. Social pollinators interact with both the air and the ground to create a unique ecology that allows toxicologists to investigate multiple exposure sources using a single species [17]. In addition to exposure routes, social pollinators accumulate metals in their hives resulting in variable exposure by age and caste [18, 19]. Exposure to some metals has shown a marked decrease in native bee populations in contaminated areas as well as low fecundity [20, 21].

Other metals such as nickel, selenium, and aluminum have been studied in the context of pollinator toxicology. Selenium affects development and lifespan, and reduces long-term memory and responsiveness to sucrose [22–24]. Aluminum similarly bio-accumulates in pollinator species and has been shown to decrease foraging adaptability without causing any taste aversion however, memory and responsiveness to sucrose stimulation have not been tested [18, 25, 26]. Aluminum is the third most abundant element in the earth’s crust but is more of a toxicological concern presently as a result of poor mining practices and acidification caused by human activity [15, 27]. Acidification from anthropogenic emissions is a global risk, but is of most concern in eastern North America, northeastern South America, central Africa, and the southeast Asian Islands [28, 29]. Acidic soils contain ionized forms of otherwise bound metals; the ionized molecules are more bioavailable and less likely to be excreted [16]. This is of particular concern with aluminum as it has been shown to accumulate in ganglia when ionized and has been linked to neurodegeneration, reduced population growth in invertebrates, and bio-accumulation in social insects [18, 20, 21, 30–32].

Reported values of aluminum in North America in produce and pollen range between 0.05-670mg/L [33]. Acidification is not the only risk however; concentrations between 10.4 and 268mg/L have been found in pollen near Bauxite mined areas in Brazil [34]. Bauxite mining is the process through which aluminum is extracted from the soil and is the second primary route through which contamination can occur. The mining process produces acidic waste products that contaminate the surrounding areas. This is the most likely contributor to increased aluminum concentrations in soils in South America and Australia [27, 34]. Considering such a wide range of contamination levels, it is important to understand how exposure, especially in highly contaminated regions, may affect bees.

### Honey bee risks

Aluminum contamination is likely a global issue with exposure levels dependent on local environmental conditions [28]. As honey bees have dispersed globally, subspecies have diverged with differential responses to pathogens, foraging problems, and likely susceptibility to toxicant exposure [35–37]. Honey bee decline is not occurring at the same rate worldwide and likely subspecies factors contribute to the regional effects [4]. New subspecies of honey bees are discovered fairly regularly (see [8, 38, 39] for examples) implying that the species readily diverges and becomes reproductively isolated [40]. Here we use two allopatric species, *Apis mellifera carnica* and *Apis mellifera caucasica*, to understand how subspecies with similar ecosystem variables respond to aluminum exposure and compare them to a relatively spatially diverse species (*Apis mellifera mellifera*). The global occurrence of *Apis mellifera mellifera* is due to human transport over the last several hundred years [41]. Previous research suggests that allopatric subspecies may have similar forage collection however, we do not yet know how their tolerance to toxicant exposure may differ [37]. Understanding how toxicants may affect subspecies differently may help us to understand which subspecies are the most at risk and which regions should focus on toxicant reduction.

### Neural mechanism

The neurochemical mechanism through which aluminum is expected to act occurs throughout honey bee subspecies and is conserved even through mammalian physiology [42]. It is hypothesized to bind to the cholinergic enzyme acetylcholinesterase (AChE) which is the degradation enzyme for acetylcholine [13, 43, 44]. The effect on the cholinergic system works similarly to neonicotinoids. Binding of aluminum to AChE can cause temporary hyperkinesia, memory loss, spasms, and eventual death [42, 45]. These consequences are expected in honey bees and likely other pollinator species. Lipid peroxidation from malfunctioning cholinergic systems may also contribute to neurodegeneration as this process can produce excess reactive oxygen species which may degrade brain tissue [13, 45, 46]. Aluminum has also been reported to disrupt the insulin hormone system, which may inhibit accurate food quality decisions [47, 48]. It is important to understand how these mechanisms may affect behaviors important to forage and mobility and to determine if aluminum can cause death at known pollen concentrations.

### Behavioral change

Eusocial bees rely on successful food collection not only for individual survival but to provide sustenance to non-foraging hive-mates and to develop winter stores. Reduced foraging skills or disrupted flight patterns from metal exposure may therefore cause hive-wide poor health. Free-flight studies allow us to determine if exposure is realistically altering pollinator forage and if these effects may be a concern. Previous behavioral experiments have found that there is likely little to no taste aversion from aluminum in nectar and that there may be foraging deficits from exposure [25, 26]. These studies have shown that aluminum affects floral color fidelity, and that visitation duration and frequency may change dependent on exposure concentration. These results occur almost immediately after exposure to low aluminum concentrations in nectar [25]. However, there is a gap in the literature regarding disorientation, motor control, and hyperactivity, or if color bias is dependent on the attractiveness of the color choices offered. We employ free-flight and laboratory studies to look at these variables in environmentally relevant (free-flight) and highly controlled (captive laboratory) conditions.

In addition to disorientation and hyperactivity, bees may choose poorer resources after exposure to aluminum. Honey bee species in Turkey showed a preference for blue flowers over white when the flowers contained equal rewards. However, when flowers contained unequal rewards, and bees were exposed to aluminum, they did not differentiate higher value flowers based on color cues [25]. There is debate as to whether a comparison between blue and white flowers or blue and yellow flowers may show starker evidence of color preference. When offered the choice between blue and yellow flowers, honey bees may actively visit only one color morph whereas when offered blue and white flowers bees typically visit both colors with a slight blue preference [35, 49]. As a previous study showed a slight depression in color preference when exposed to low doses of aluminum, the present study uses yellow-blue comparisons to attempt to further unravel preference change as a result of exposure [25].

Honey bees rely in-part on color cues to follow the environmental changes throughout the flowering season. As bees exit the hive and begin foraging the first visitation elicits a lasting color preference for the entirety of their foraging life, approximately 1 week [50, 51]. This preference reflects the general flowering environment in which a bee will spend her foraging time. However there is evidence that the preference can be flexible dependent on floral environment and genetic diversity [51, 52]. If aluminum exposure reduces the flexibility of color choice this could be maladaptive for hive-wide feeding and establishing winter stores as bees may be selecting the less calorically valuable flower patches. As floral environment changes, bees must be able to focus their visitation on the highest caloric benefit rather than visiting many low quality resources.

In addition to correct floral choice, time spent to find and collect food resources should be optimized to increase the food stores of the hive. Although a previous study used a portion of total time to estimate this parameter, the results were highly variable and did not account for individual variation [25]. The current study will use video recordings to determine the exact time between visits by individual bees. This will be a more accurate depiction of whether disorientation is occurring.

Aluminum may cause hyperkinesia, spasms, and death when exposed to high concentrations as a result of the expected decrease in AChE activity [31, 53]. Hyperkinesia may affect how honey bees manipulate floral rewards to access nectaries and their ability to contribute to the hive. Flowers typically contain reproductive parts that must be manipulated to access the food resources. Floral manipulation is part of a cost-benefit analysis by bees to determine which flowers are the highest quality [54]. The current study will determine if hyperactivity immediately affects forage and how activity is affected by chronic aluminum exposure by looking at drinking time and interactions with the free-flight cap pushing apparatus and activity across the captive lifespan.

In addition to prolonged exposure causing premature death, hyperactivity may reduce the time spent resting. Foraging bees have strong diurnal circadian rhythms that are timed with flowering [55, 56]. Disruption of this cycle due to hyperactivity may limit their access to high-quality food resources as they are not foraging during peak bloom. Their activity may be increased but with poor circadian cycling that may not translate to increased foraging output. Hyperactivity from over-stimulation of cholinergic receptors may also have consequences outside of foraging deficits. Previous study has not yet developed a toxicity curve for aluminum in pollinator species so we do not know if concentrations found in pollen are immediately lethal or how chronic exposure may affect bees. We expect based on previous aluminum research, that honey bees will be disoriented, have poor choice making skills, be hyperactive, and be arrhythmic.

The methods used here can determine how circadian rhythms, mobility, survival, and flight variables are affected by aluminum ingestion in allopatric and non-allopatric subspecies of honey bee. We expect decreased circadian rhythmicity and increased motility and mortality. In a free-flight experiment, we expect lower return rates as a measure of disorientation and increased errors as a measure of poor foraging choice. These studies will be a comprehensive examination of the outcomes of exposure to aluminum as well as a description of new techniques that can be used in invertebrate toxicology.

## Materials and methods

The effects of aluminum were tested using two experimental paradigms. The first, a free-flight cap-pushing experiment, compared two subspecies of honey bee *Apis mellifera mellifera* (OK-M) and an *Apis mellifera mellifera/ scutellata* hybrid (PR-S) from Oklahoma, USA and Puerto Rico, USA, respectively (Table 1). The cap-pushing experiment investigated how floral manipulation and disorientation are affected by aluminum as well as color preference when blue and yellow flowers are available. The second experimental paradigm, the monitor system, examined how aluminum affects motor activity, circadian rhythmicity, and survival. The monitors are a laboratory-based incubator experiment and included three subspecies of honey bee, OK-M, *Apis mellifera carnica* (T-Car) and *Apis mellifera caucasica* (T-Cau, Table 1). The latter two subspecies are allopatric and indigenous to the Mediterranean and were at a common apiary in Tekirdağ, Turkey.

**Table 1:** Description of subspecies included in each experiment, when the experiment was conducted, doses applied, and locations.

Sub species Name	Abbreviation	Experiments	Location of Experiment	Longitude and Latitude	Concentration of Al Used (mg/L)	Ingested Dose of Al
*Apis mellifera mellifera*	**OK-M**	Cap Pushing, Subspecies Toxicity, Dose-Response Curve	Stillwater, OK USA	36°06'19.9"N 97°02'56.1"W	10.4, 20, 25, 40, 75, 134, 201, 268*	Variable
*Apis mellifera mellifera/ scutellata*	**PR-S**	Cap Pushing	Gurabo, PR USA	18°15'26.2"N 65°59'12.0"W	40**	1.996μg
*Apis mellifera carnica*	**T-Car**	Subspecies Toxicity	Tekirdağ, Turkey	40°59'30.6"N 27°34'40.1"E	40**	1.996μg
*Apis mellifera caucasica*	**T-Cau**	Subspecies Toxicity	Tekirdağ, Turkey	40°59'30.6"N 27°34'40.1"E	40**	1.996μg

Bee abbreviations refer to both the location of experiment and the subspecies name for ease of reference.

*Range selected from [22], intermediate dosages selected to show a range of outcomes.

**40mg/L selected based on a low to intermediate concentrations from [22].

As the subspecies are not the same between the two experiments OK-M will be used as the primary comparative subspecies. OK-M are a globally present subspecies as a result of human relocation and are often the main focus of honey bee study [41]. This makes OK-M the ideal subspecies for comparison for both studies. All bees were foragers, assumed to be ≥20 days old [57]. Subspecies of honey bee are extremely similar in morphology but have been shown to demonstrate behavioral differences and may have different responses to toxicants [35, 36, 58, 59]. PR-S bees are a genetically unique hybrid of Africanized bees (*Apis mellifera scutellata*) and European bees (*Apis mellifera mellifera*) likely as a result of island selection [59]. The unique hybrid is docile like European bees making them safe to work with but with the ability to resist *Varroa destructor* similar to Africanized bees. Their unique behaviors may also indicate distinct toxicological responses as compared to their parent subspecies.

### Aluminum concentrations and rationale

Tap water was primarily used for these experiments as that was what was available in the field. Both control solutions and experimental solutions were made using the same source of water throughout each experiment. For the monitor experiments in Oklahoma, USA filtered water was used as it was available and could minimize possible outside contaminants.

Aluminum was administered to bees as AlCl_3_ in an aqueous sucrose solution or as AlCl_3_ in water. The experiments described use aqueous concentrations ranging from 10.4mg/L to 268mg/L which represent the range of aluminum concentrations found in pollen in Brazil [34]. Although we use water as the primary solvent for AlCl_3_, we are not trying to make comparisons to contaminated water sources as aluminum concentrations in waterways do not typically rise above 1mg/L. [60, 61]. We cannot directly replicate pollen consumption in a short-term experimental setting, for this reason, we use pollen and plant tissue-based concentrations to approximate nectar concentrations [32, 33]. Previous investigation into metal uptake and distribution to pollinators suggests that although pollen concentrations can be higher than nectar concentrations, nectar is likely contaminated similarly [62]. The cap pushing experiments used a relatively low concentration compared to what has been found in pollen and could be expected to occur in nectar (40mg/L). The comparatively low value was selected to avoid hive exposure from regurgitation and possible metal accumulation in the hive. However, this concentration is higher than the lowest concentrations found in Brazilian pollen to increase the likelihood that an effect could be detected [19, 33].

For the monitor experiments, the bees are captured and cannot expose the entire hive through regurgitation. For this reason, bees were given concentrations that mimic higher nectar concentrations delivered through their water source. There was physical separation between their carbohydrate and protein source from their water source. Bees were incubated at 35°C. Previous research on water collection in bees suggests that at this temperature a relatively low amount of water is collected by bees, this likely translates to limited water usage [63]. The concentrations used may be high for nectar, however as a result of limited water use, the actual water ingestion and therefore toxicant ingestion rates are expected to be low. This low ingestion should result in a fairly conservative metric of exposure outcomes.

### Cap pushing paradigm

The two experiments were run in different time periods and locations. *Apis mellifera mellifera* (OK-M) experiments were run between August and November 2016 and *Apis mellifera scutellata* (PR-S) experiments occurred during July 2017. All bees were trained to manipulate caps following methods originally presented in Abramson et al. [64].

Honey bees were trained to visit a training station with a 1M aqueous sucrose feeder. After the feeder was consistently visited by approximately 10 bees it was removed and replaced with a black 3-D printed platform (27cm x 8cm x 1cm) containing two equally-spaced wells. Ten bees was chosen to switch from the feeder to the platform as it was determined to be the limit at which more visiting bees did not increase the likelihood of successful platform visitation. Each platform well was filled with approximately 100μL of 1M aqueous sucrose. After approximately five bees were consistently visiting the platform, returning bees were marked with Testor’s paint (9115X, Vernon Hills, IL) for identification. After painting, the platform was switched out for an identical platform, this time with one well containing 50μL of 1M aqueous sucrose covered by a white partially-enclosed cap while the other well was left empty and uncovered (see Table 2 for procedural details). After a bee successfully moved the cap and drank the reward, the platform and cap were switched out for an identical setup. The training cap side was alternated following a paradiddle pattern (LRLL RLRR).

**Table 2:** Outline of cap pushing paradigm procedures and trip counts.

Phase	Number of Trips Required	Variables included in Results
Training Phase 1 (Cross Cap)	≥2	None
Training Phase 2 (Asterisk Cap)	≥2	None
Mastery	7	Return Rate, Correct Interactions
Treatment (Mastery Cap Used)	1	Return Rate, Correct Interactions, includes 15 minute hold period
Painted Color 1	12	Return Rate, Correct and Incorrect Interactions, Initial Color Choice
Painted Color 2	12	Return Rate, Correct and Incorrect Interactions, Initial Color Choice

**Fig 1:**
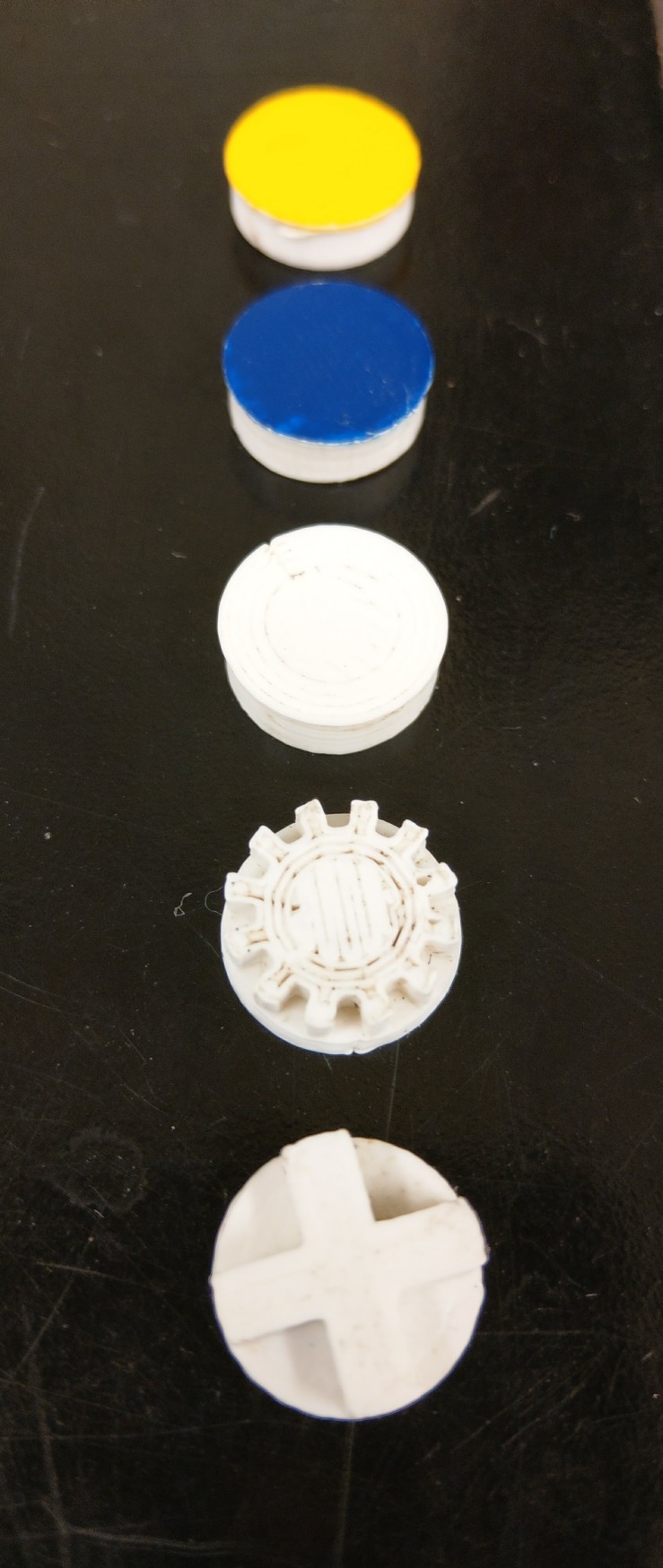
Examples of (from bottom up) training, mastery, and painted caps. View from the underside of caps for training caps. Training caps look identical to mastery cap from the top angle.

For the training phase of the experiment, two 3-D printed training style caps were used. The first training cap had a T-shaped base for easy access to the sugar well. The second training cap was an asterisk-shape which reduced the well accessibility further (Fig 1). After two visits to each of the training caps, the mastery cap was introduced. The mastery cap has fully enclosed sides and requires a full push to access the sucrose well. Each cap is 1.5 cm in diameter, 0.5 cm tall, and weighs 450±50 mg with a solid white top to maintain a constant image when flying in from above.

**Fig 2:**
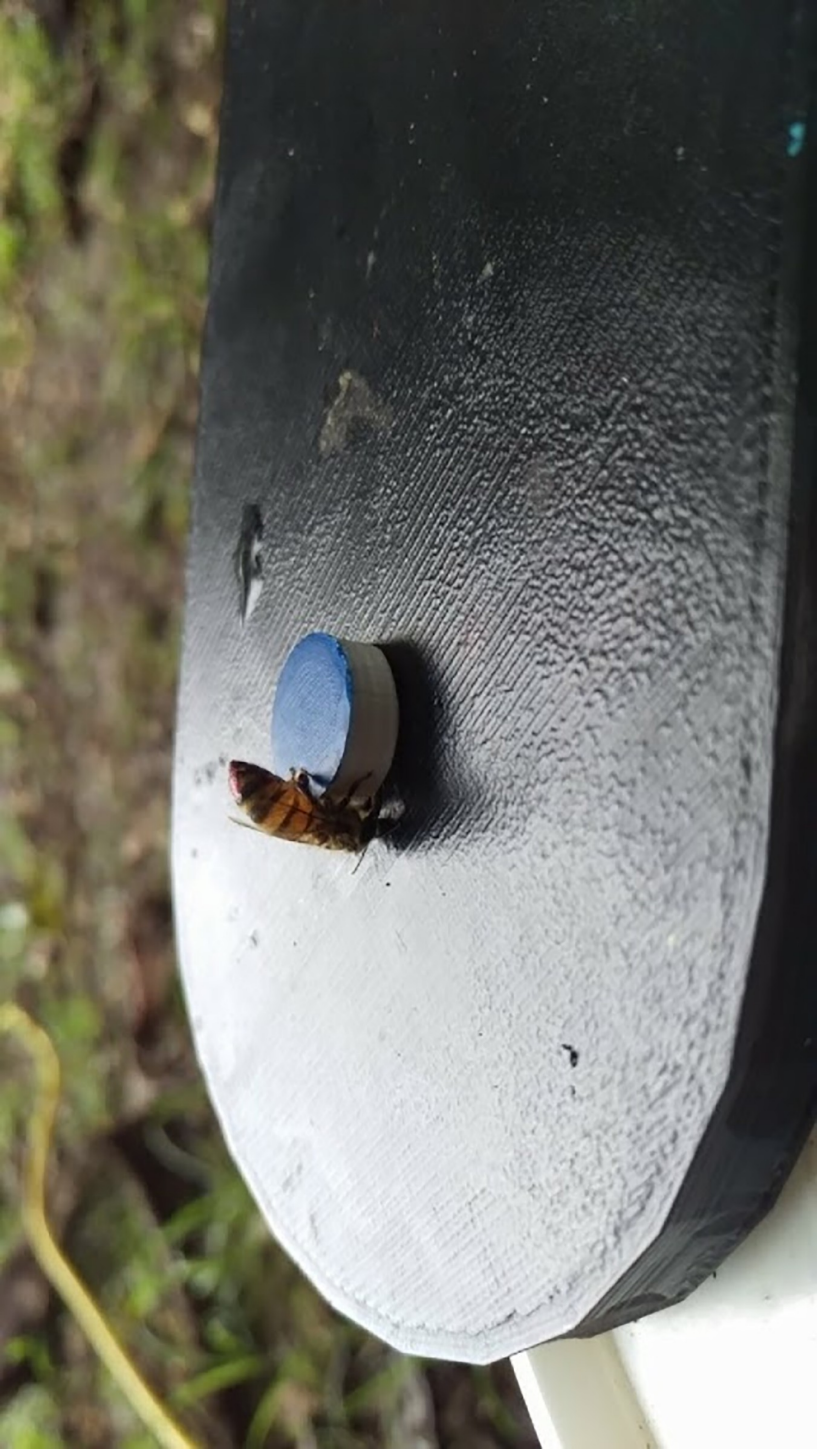
Trained honey bee pushing fully enclosed painted cap.

During the mastery cap phase, a video camera was set up to record the remaining experiment. For each bee, 7+1 (Table 2) mastery cap trials were completed. OK-M bees were only video recorded for 4–6 visits and PR-S bees were recorded for all eight visits of the mastery phase. On the eighth visit, treatment was placed underneath the well. Treatments were either 50μL of 40mg/L Al (1.996μg Al) in 1M aqueous sucrose or 1M aqueous sucrose. A small bell jar was placed above the bee so as not to disturb her feeding and kept in place for 15 minutes. The rationale behind restraining the bee was to increase individual digestion and limit hive contamination. Before release from the bell jar, a second identical platform was placed next to the platform holding the bee. The second platform contained two caps, one painted yellow (Testor’s 1632T) and one blue (Testor’s 1208T), each covering one well (Figs 1 and 2) One well contained 1M sucrose, the other well contained water. For a bee to be included in the data set, twelve trials were completed with Color 1 covering the sucrose reward followed by twelve trials of Color 2 covering the reward (Table 2). Color 1 was randomly chosen at the start of the experiment. For an example of a honey bee successfully pushing a cap see S1 Video.

Videos were coded and analyzed for color of initial cap interaction per visit, number of correct and incorrect interactions before and after feeding per visit, platform side preferences, latency to correct side push (handling time), time between exiting and returning to the platform (return time), and time taken to drink reward (drink time). Variables that showed no differences between subspecies or by aluminum ingestion were not included in the results section. In total there were more OK-M bees (n_control_ = 10, n_40mg/L_ = 12) than PR-S bees (n_control_ = 7, n_40mg/L_ = 9).

### Monitor system

**Fig 3:**
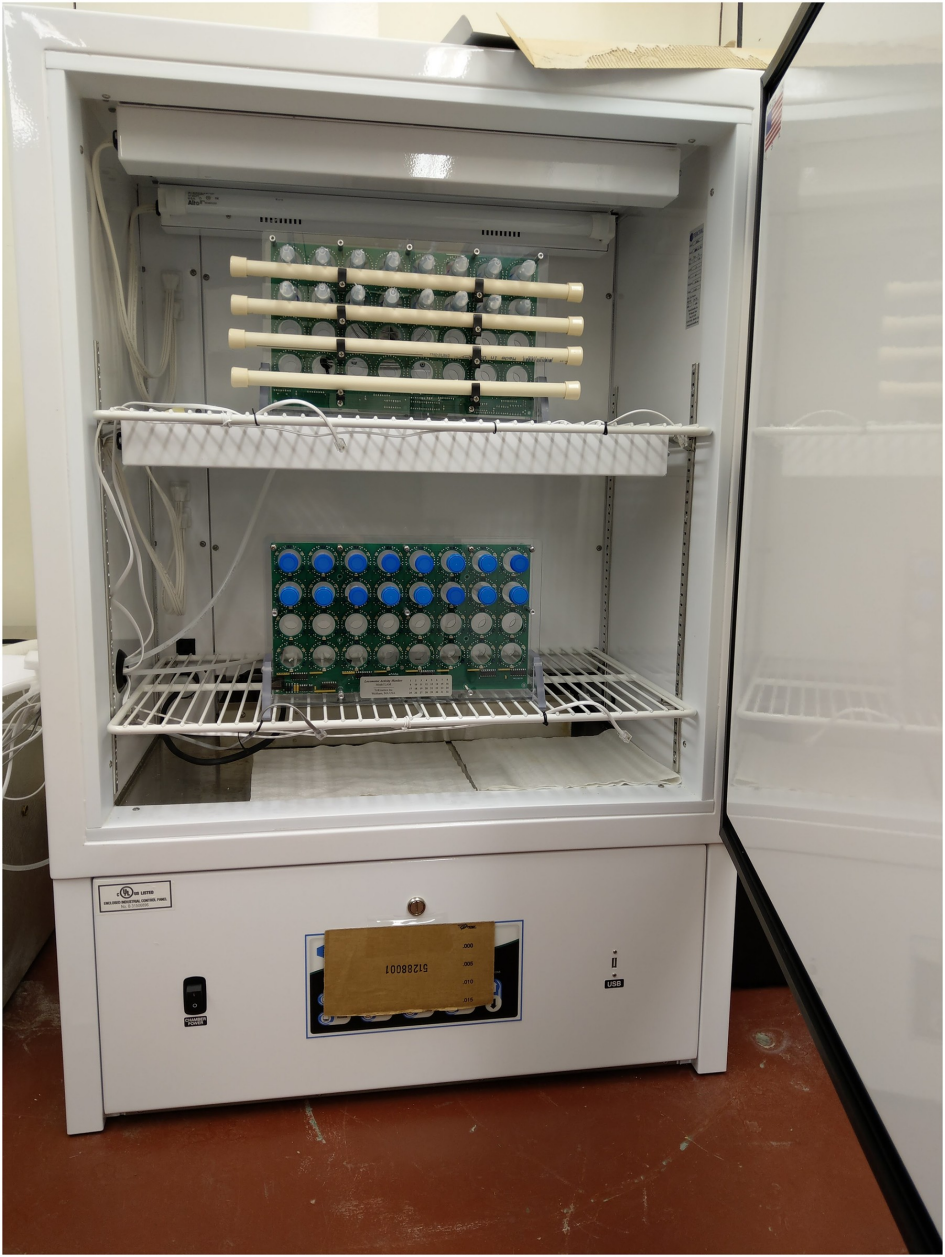
Monitor system in incubator. Top rack shows the monitor with CPVC piping which allows water or treatment access for the bees. Lower rack shows falcon tube caps containing bee candy.

Monitors (Fig 3 TriKinetics Inc. Waltham, MA) can be used to understand effects on lifespan, circadian rhythmicity, and motility of individual bees. Using this system allows us to expose bees to aluminum for longer periods to simulate chronic exposure. The apparatus also allows us to expose the bees with higher concentrations of aluminum, such as those found in pollen in Brazil, without risk to the entire hive [34].

The monitor apparatus houses up to 32 bees in individual 15mL falcon tubes. Each falcon tube contains several aeration holes. Lids of the falcon tubes were filled with a pea-sized dollop of approximately 40% honey, 60% sucrose mixture, or “bee candy”. Bee candy was covered with a piece of cheesecloth, approximately 2cm x 2cm, to limit the bees becoming adhered to the mixture while still allowing nutritive access. On the opposite end of the falcon tube from the lid, one aeration hole was fit with one end of a piece of filter paper approximately 0.25cm x 3cm. The strip of filter paper extended from the inside of the falcon tube down to a 40cm long x 1cm inner diameter piece of Chlorinated Polyvinyl Chloride (CPVC) pipe running the length of the monitor (Fig 3). The CPVC pipe was filled with up to 40mL of water before placing the monitor with bees inside an incubator. The water travels up the filter paper to each bee (8 bees/CPVC pipe). Water contained the treatment for each experiment. Concentrations presented are for aluminum only; AlCl_3_ (Sigma-Aldrich St. Louis MO, 99%) was added at 5x these concentrations to account for the weight of chloride.

The monitors contain six photocells encircling the center of the falcon tube; these are positioned along the green walls in Fig 3. The photocells record each time a bee crosses the centerline of the tube. Bees that do not cross the centerline for 24 hours are recorded as deceased. Circadian rhythms, activity level, and captive lifespan are recorded via this system. Monitors were kept in 24 hour darkness for the entirety of the experiment with the exception of water and food replacements during which the bees were exposed to red light. Bees do not have vision in the red spectrum so the red light should not influence the circadian behaviors [65]. Each CPVC pipe was filled with 40mL of water with or without AlCl_3_ at the start of the experiment. Every subsequent 48± 8 hours the CPVC pipes were filled with up to 20mL of water (as needed) by treatment. Every other water refill included a re-capping with a fresh food lid for each living bee. Exposure to red light was at least 2 hours removed from the previous water refill as a precautionary measure to limit confounds in circadian rhythm results. For example, if bees were removed at 12pm on Monday, the Wednesday refill would occur earlier than 10am or after 2pm. Control bees can live up to three weeks in these conditions.

Two experiments were run using the monitor system on three subspecies of honey bee. All bees were from 10-frame Langstroth hives and had active foraging populations as determined by the number of bees exiting the hive (>10 bees exiting per 20 second period). *Apis mellifera caucasica* (T-Cau) and *Apis mellifera carnica* (T-Car) were collected during the summer of 2016 near Tekirdağ, Turkey. T-Cau and T-Car bees were collected by covering the hive entrance with net, allowing foragers to be caught during take-off. This procedure was established to minimize mixed subspecies confounds as both T-Cau and T-Car subspecies were maintained within the same apiary and feeders could easily be contaminated with other subspecies. T-Cau and T-Car bees were then transferred to falcon tubes on-site before being taken to a laboratory and installed in monitors. *Apis mellifera mellifera* (OK-M) were collected off of a feeder in Oklahoma, USA during the late summer of 2017. OK-M bees were caught using falcon tubes and therefore did not require transfer before being taken to a laboratory. All bees were assumed to be foragers and were installed in the monitors as quickly as possible, within 4 hours of capture. Bees were randomly assigned to monitors and therefore treatment. Each monitor contained one treatment concentration and one subspecies per experimental session. For each experimental session a simultaneous 0mg/L monitor containing the same subspecies as treatment experiments was run to control for temporal and environmental factors.

Variables of circadian rhythmicity, activity level and captive lifespan were measured. Rhythmicity index (RI) is a coefficient that can be used to estimate their daily sleep-wake cycle. Mean activity is the average activity per hour per bee averaged across total bees in the monitor. Mortality was recorded from an actogram and number of captive days alive includes day of death for each bee. To standardize rhythmicity and activity, Eqs 1 and 2 were used for each treatment and location. Data was compiled in MatLab (R2014b) before being analyzed in JMP Pro 13.

$$\frac{\frac{{rhythmicity}_{bee(1)}}{{daysalive}_{bee(1)}}+\frac{{rhythmicity}_{bee(2)}}{{daysalive}_{bee(2)}}+\frac{{\ldots rhythmicity}_{bee(n)}}{{daysalive}_{bee(n)}}}{n_{bee}}$$ (1)

$$\frac{\frac{{activity}_{bee(1)}}{{daysalive}_{bee(1)}}+\frac{{activity}_{bee(2)}}{{daysalive}_{bee(2)}}+\frac{{\ldots activity}_{bee(n)}}{{daysalive}_{bee(n)}}}{n_{bee}}$$ (2)

#### Subspecies toxicity

Three subspecies (*Apis mellifera mellifera* (OK-M, n = 32), *Apis mellifera carnica* (T-Car, n = 32), and *Apis mellifera caucasica* (T-Cau, n = 32) were run to determine how activity levels, circadian rhythmicity, and mortality differed at 40mg/L Al. This concentration was chosen to reflect the nectar in moderately acidified regions that cover wide swaths of eastern North America, northern South America, central Africa, and Europe [28]. Although these regions are not the highest risk areas, they represent regions where bees are economically valuable. If there are consequences at this relatively low concentration then there may be cause for concern at a much greater scale than simply within the extremely acidified regions.

Treatment solutions (water, water with 40mg/L Al) were made at the start of each experiment. T-Cau and T-Car honey bees were run in monitors near Tekirdağ, Turkey. Due to facilities restraints, treatment solutions were not refrigerated for these experiments. T-Cau and T-Car bees were kept at average temperature and humidity of 35°C and 77% respectively. This is a similar temperature and humidity to an average hive [66]. OK-M bees were run in monitors in Stillwater OK, USA. These bees were given refrigerated solutions to minimize bacterial growth. The incubators in the Stillwater laboratory could not maintain the humidity level of the Turkish facilities without condensation and bees were kept at an average humidity of 42% at 35° C.

#### Dose-response curve for *Apis mellifera mellifera*

Two experimental sessions were run to determine how increasing concentrations of aluminum in water effects *Apis mellifera mellifera* (OK-M). Sessions were confined by the number of monitors available (4) and were split into high dose (October 3- October 23, 2017) and low dose (October 23-November 20, 2017). Both sessions included a control (0mg/L) monitor. All solutions were made using deionized water. The first experimental session contained 24 OK-M per monitor at concentrations of 0mg/L, 134mg/L, 201mg/L, and 268mg/L aluminum. The second experimental session included 24 bees per monitor of 0mg/L, 10.4mg/L, 25mg/L, and 75mg/L aluminum. The controls were compared via T-Test (t_captive days alive_(24.61) = 1.37, p = 0.18, t_rhythmicity index_(39.96) = 0.711, p = 0.48, t_activity_(40.61) = 1.2, p = 0.24) to determine if temporal variables significantly affected the results. The two session controls were not significantly different, therefore temporal effects were minimal and controls were grouped. Solutions were made fresh before each experimental session and were kept refrigerated at 4°C for water refills throughout the experiment. Concentrations of 134mg/L and 201mg/L were made from serial dilutions of 268mg/L whereas 10.4mg/L and 25mg/L were made from serial dilutions of the 75mg/L solution. Bee candy was refrigerated but discarded after 10 days and remade as needed.

## Results

### Cap pushing paradigm

**Fig 4:**
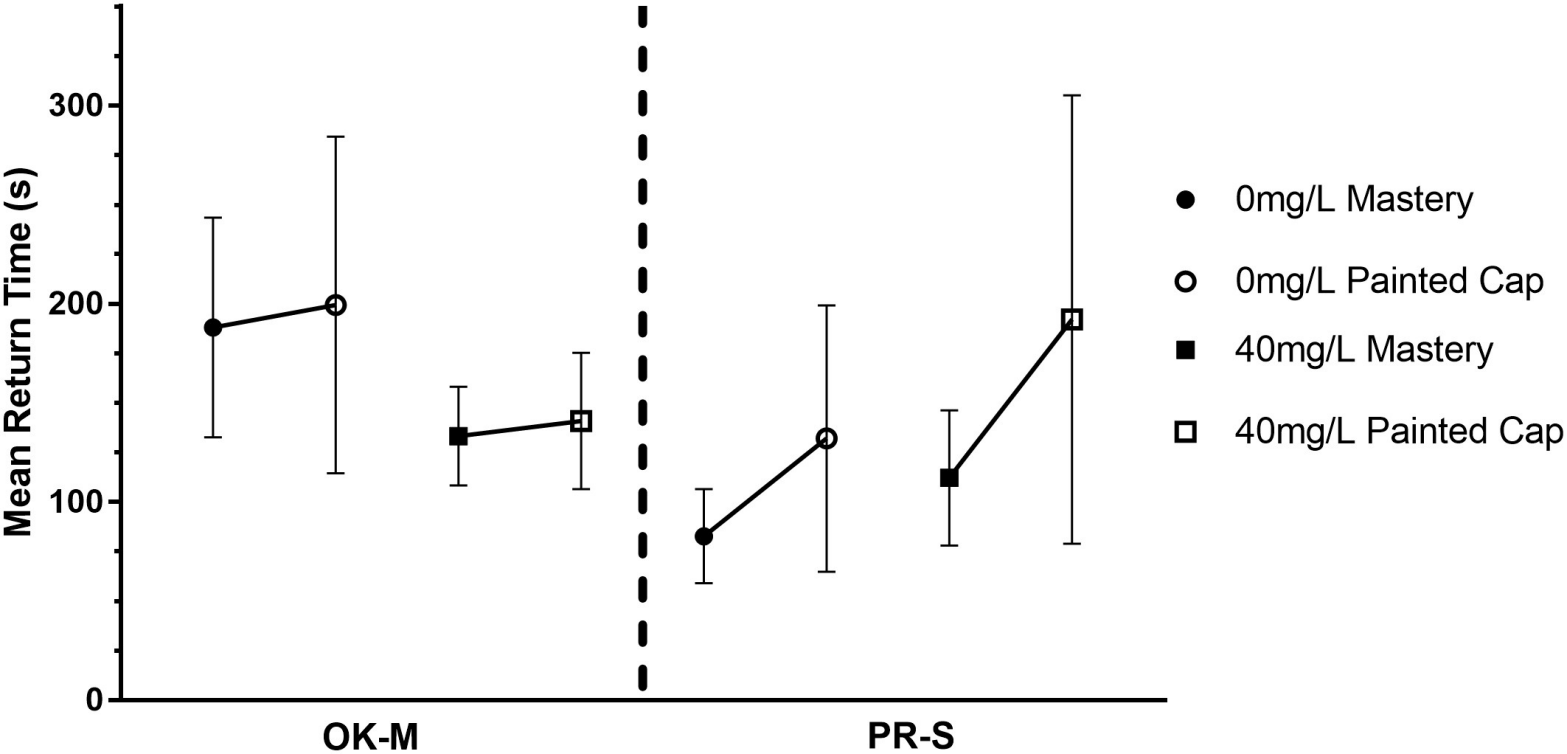
Mean time to exit the platform and return for another visit by subspecies and concentration. For each exposure concentration the right-most point is the mean before exposure (4–8 platform visits) and the second point of the mean after exposure (24 total platform visits). Error bars represent the standard error of the mean.

Bees were expected to have increased return rate and increased color choice errors after aluminum exposure as compared to bees that were not exposed. Bees were highly variable in their return time. This variability increased for all subspecies and concentrations between mastery cap and painted caps with no significant difference between or within subspecies (Fig 4). In both locations bees were more variable in the post-treatment phases however this effect is universal and is therefore not an effect of the aluminum.

**Fig 5:**
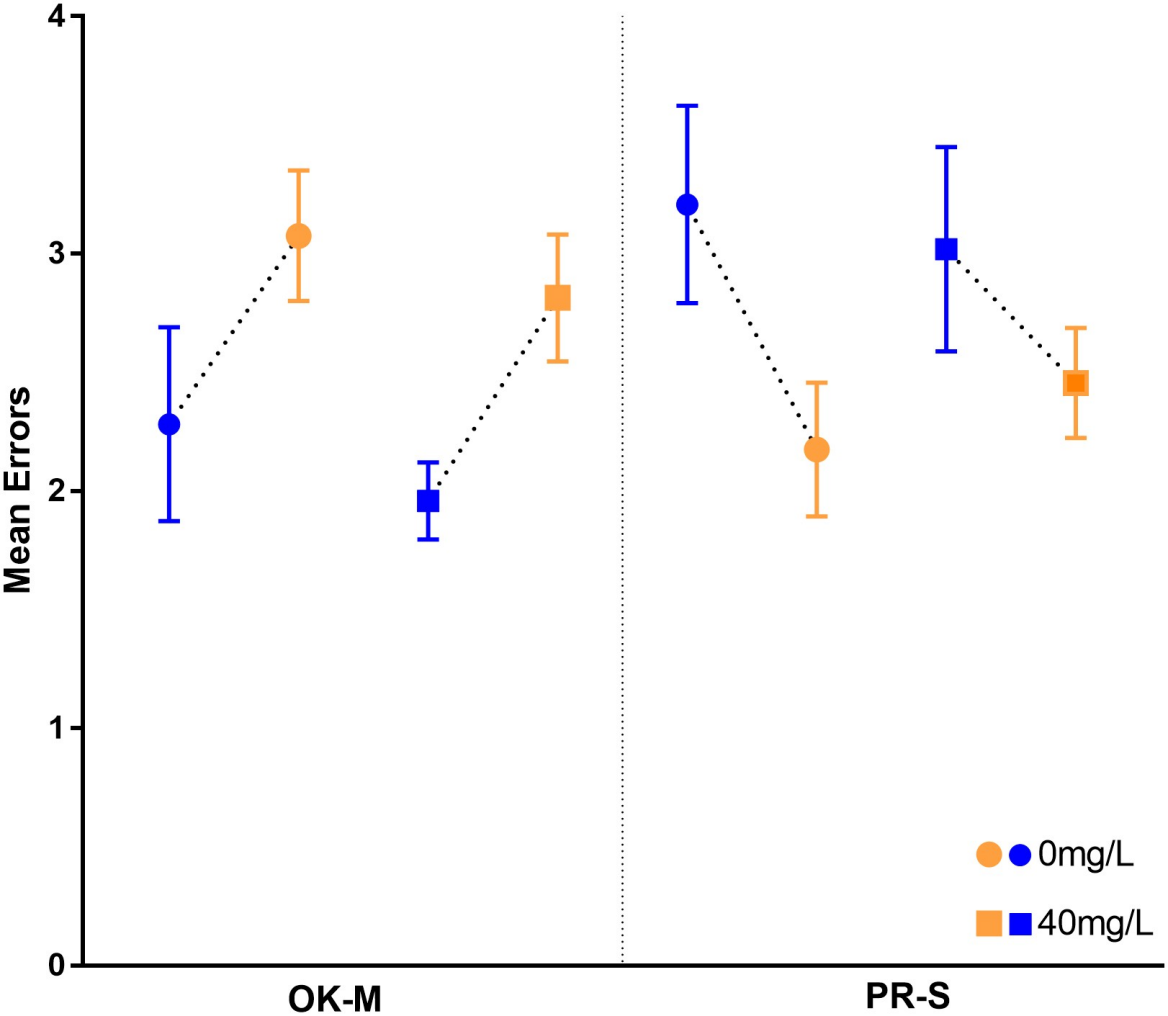
The mean number of errors by exposure concentration and subspecies. Errors were defined as touching the cap covering water. The presented data includes interactions before and after the reward was consumed. Blue points represent when blue caps covered the reward and yellow points represent when yellow covered the reward.

The number of errors was expected to increase in bees that had been exposed to aluminum as compared to controls. There was not a significant difference in the total number of errors within subspecies however; the two subspecies have opposing trends (Fig 5). OK-M (see Table 2 for subspecies abbreviations and information) show significantly more errors when the yellow cap covered the reward regardless of treatment (χ^2^_0mg/L_ (11, N = 227) = 27.43, p = 0.004, χ^2^_40mg/L_ (8, N = 288) = 15.69, p = 0.047), meaning they prefer the blue cap. PR-S have the opposite response to blue caps and have more interactions with the incorrect yellow caps when blue covers the reward, however these data are not significant.

**Fig 6:**
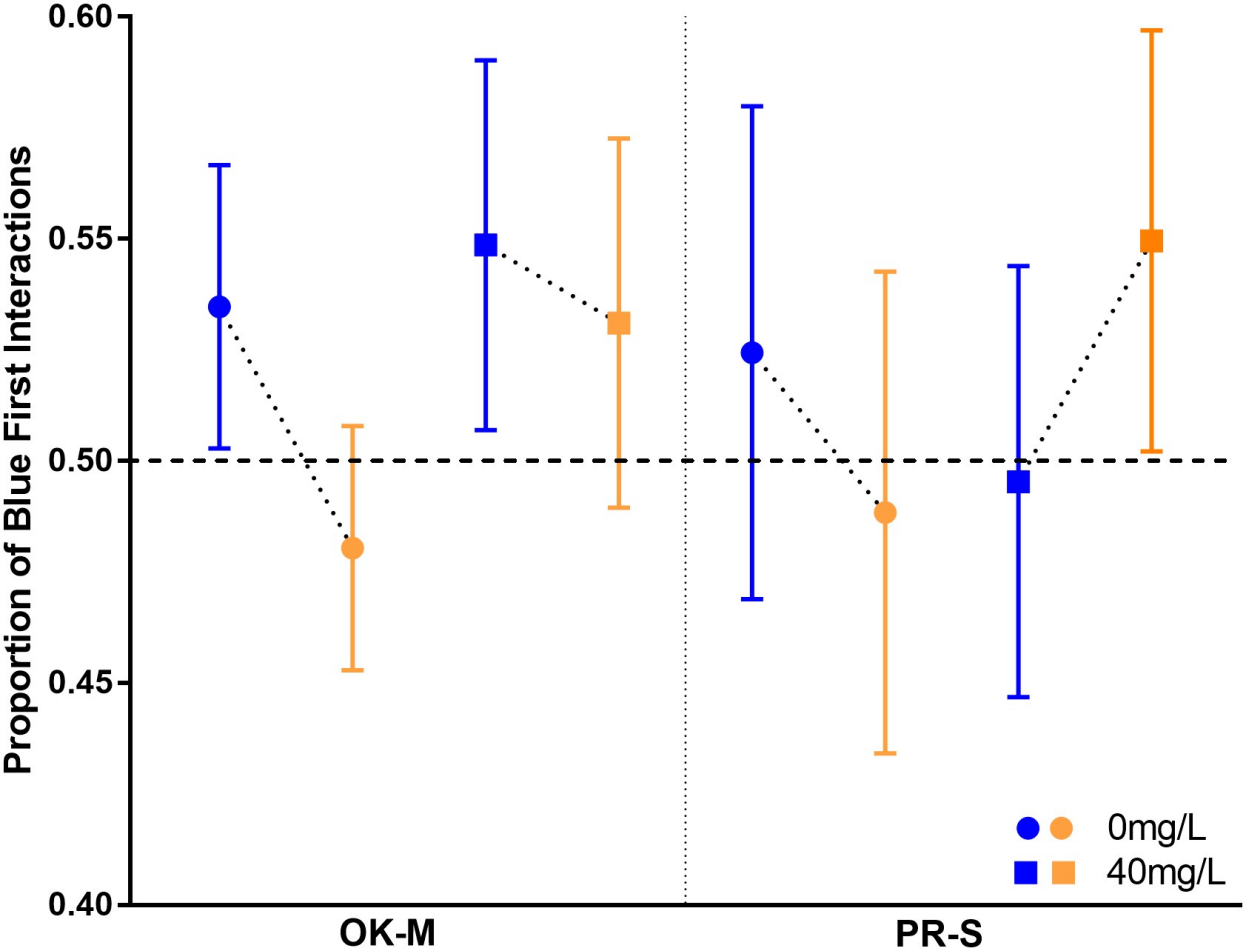
Proportion of blue first interactions by subspecies and concentration. Blue points represent when blue caps covered the reward and yellow points represent when yellow covered the reward. The horizontal line is at chance (50% likelihood of choosing either color), there was no significant variation from this point. Error bars represent the standard error of the mean.

The percentage of blue interactions is expected to be significantly above 50% when blue caps cover the reward if there is a definitive preference for blue flowers (Fig 6). However, bees did not vary significantly from random choice regardless of exposure or location. There is a trend to decrease visitation of blue caps when yellow caps covered the reward for all bees except in aluminum exposed PR-S bees. These bees increased their visitation of yellow flowers when blue flowers contained the reward.

### Subspecies Toxicity

**Fig 7:**
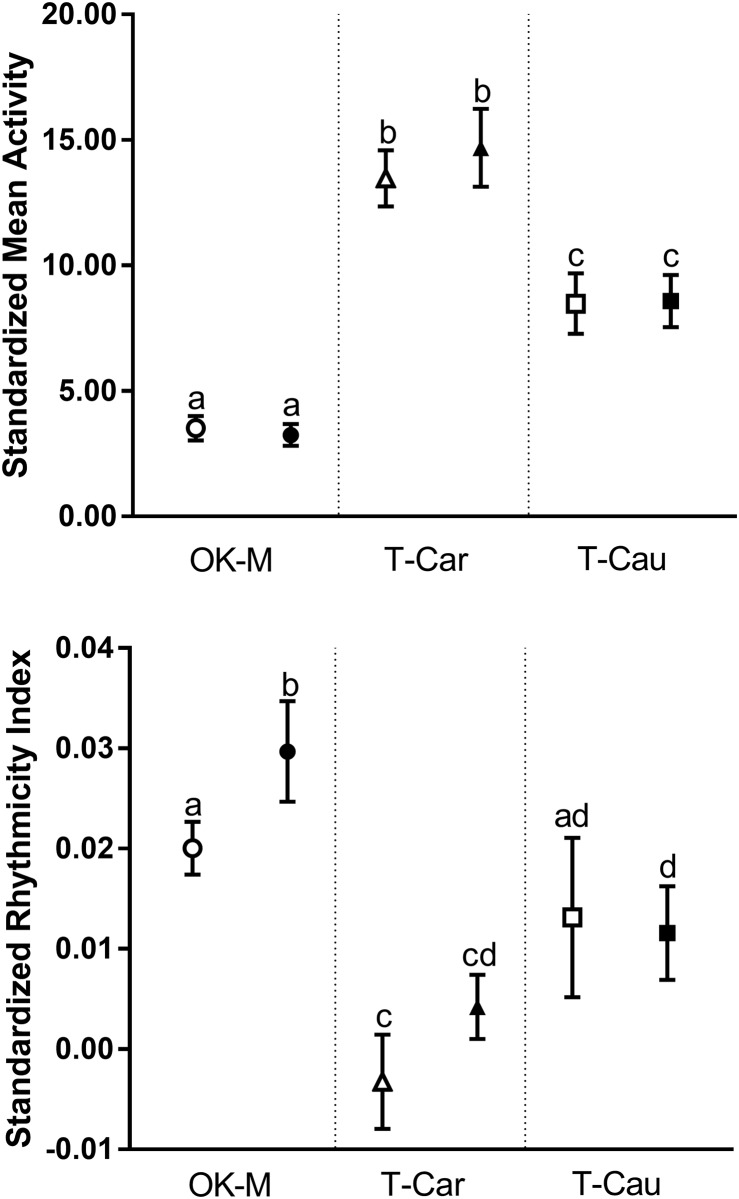
Activity level and rhythmicity index standardized by the number of captive days alive per bee. Three subspecies of honey bee are included, open symbols are 0mg/L and solid are 40mg/L. All error bars are standard error of the mean and different letters represent significant differences (p<0.05).

Subspecies are expected to respond differently to aluminum ingestion as a product of their separate evolution. Two primary responses are reported as measures of toxicity; average activity and circadian rhythms. Rhythmicity and activity data for each bee was divided by the number of days since capture before analysis to account for short lived bees containing less overall data than longer survivors (Fig 7). Analyses of variances (ANOVA) showed that there were both subspecies and toxicant effects in mean activity (df = 2, 141 F = 53.92, p<0.0001) and rhythmicity (df = 2, 141 F = 7.12, p = 0.0011). Tukey’s post-hoc analysis showed activity was different between subspecies regardless of treatment (p_0mg/L comparisons_<0.0001, p_40mg/L comparisons_<0.01) however there were no differences within subspecies. ANOVA's with post-hoc Tukey’s HSD also showed that rhythmicity was significantly different at control doses (all comparisons p<0.01) between subspecies other than OK-M and T-Cau bees. However aluminum exposure changed this dynamic and the allopatric subspecies were no longer significantly different from each other but were both significantly lower in rhythmicity than OK-M (p<0.01). The only significant difference from controls within subspecies occurred in OK-M with an increase in rhythmicity index when exposed to aluminum (ANOVA, df = 1, 55 F = 4.03, p = 0.049, Fig 7).

T-Cau showed significantly lower mean activity levels but somewhat higher rhythmicity indices than their allopatric sister subspecies. Neither T-Cau nor T-Car subspecies differed from their controls in any of the studied metrics as a result of ingested aluminum at the 40mg/L dose. Rhythmicity index is a numerical coefficient but it describes the level of adherence to a circadian cycle, time spent resting followed by time spent active in a repetitive pattern as opposed to random activity and rest. OK-M were significantly more rhythmic than other subspecies regardless of dose and showed an increase in rhythmicity after aluminum exposure implying differential toxicity. Similarly, each subspecies responded differently to the monitor system in activity level regardless of toxicant ingestion, implying variable environmental response by subspecies.

### Dose-response curve

Toxicity curves for ingested aluminum have not been established in the literature. Using the monitor system, the highest concentrations of aluminum that have been found in pollen were given to bees without risk of exposing the entire hive. The same metrics were compared as in the subspecies experiments. Aluminum is expected to temporarily increase activity level due to its cholinergic effects. However, this hyperactivity is expected to shorten lifespan. We expect that rhythmicity will decrease as bees are over-active rather than resting in a cyclical pattern.

**Fig 8:**
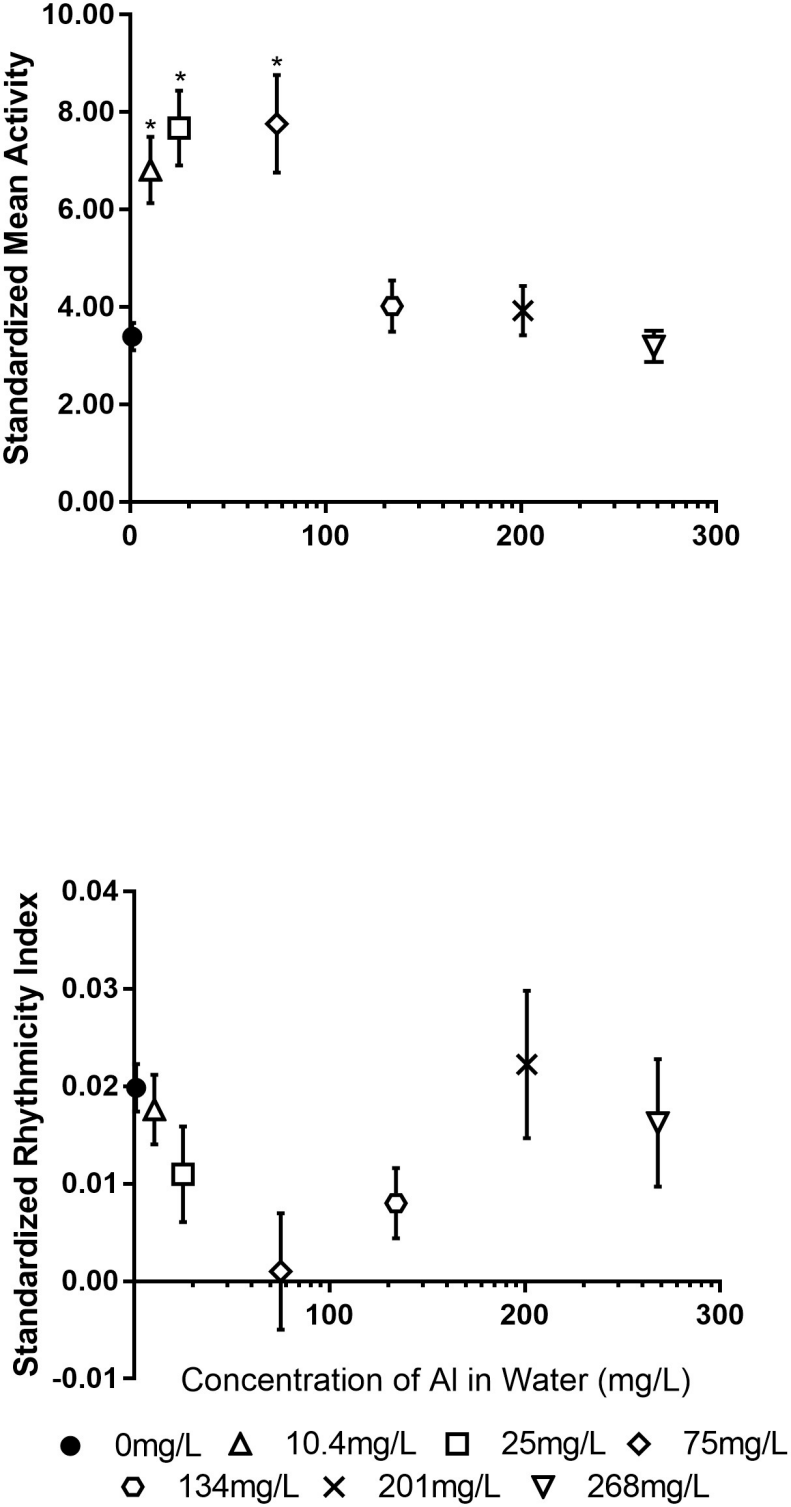
Standardized mean activity and rhythmicity at increasing doses in OK-M. Rhythmicity and activity have opposing trends. Error bars represent standard error of the mean. Asterisks represent significant difference from the control mean (p<0.05).

Aluminum had opposing effects on activity and rhythmicity (Fig 8). An ANOVA with Tukey’s post-hoc analysis showed the lowest three doses significantly increased activity as compared to controls (df = 6,185 F = 12.2316, p<0.0001 for all significant comparisons). However, this trend decreases with dose. We expect that this is a result of a hormetic response to aluminum or an decrease in acetylcholinesterase enzyme activity with high doses. This hyperactivity is also reflected in the rhythmicity data.

**Fig 9:**
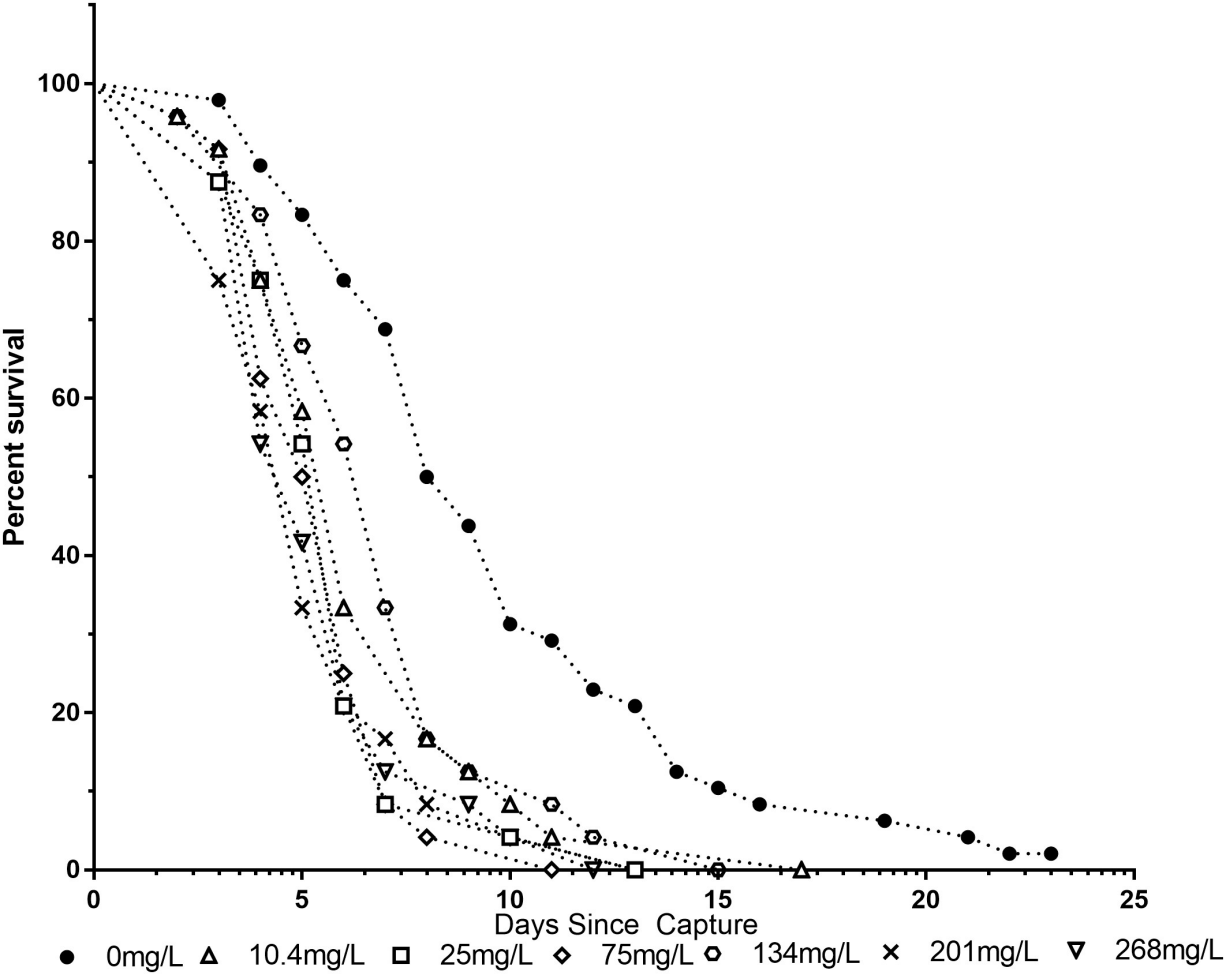
Mean number of living bees per concentration in OK-M bees. Honey bees that were not exposed to aluminum survived significantly longer than all exposed bees at all concentrations (p<0.0001).

Survivability was also affected by aluminum exposure (Fig 9). All doses significantly (p<0.01) reduced survival as compared to control using a Log-Rank test with Holm-Sidak adjustment for multiple comparisons. This implies that any exposure to aluminum affects honey bee lifespan.

## Discussion

Aluminum does not immediately limit foraging capabilities at low doses. It does not cause immediate disorientation and may only minimally change color preference in some subspecies of honey bee. Aluminum does however drastically affect OK-M bees in lifespan, circadian adherence, and motility at the concentrations used in this study. This subspecies of honey bee occurs in two regions where population decline has been a concern; North America and Europe [4]. The OK-M subspecies may be at severe risk as a result of acidification combined with stressors such as pathogens and food limitation and this may be cause for further investigation of aluminum toxicity [4, 30, 34].

Aluminum does not seem to cause significant immediate foraging deficits from incorrect color choice or disorientation in the two subspecies studied. These subspecies are fairly recently diverged and could still have similar toxicological effects [59]. Puerto Rican (PR-S) subspecies do have different color preferences compared to the Oklahoman (OK-M) subspecies. Despite the lack of statistical significance as compared to random choice, the PR-S results could be the beginning of a stronger trend to abandon their slight yellow preference when exposed to aluminum. These results imply opposing preferences between the subspecies which may be affected by toxicant exposure differently. We expect that this trend would be significant using higher aluminum concentrations such as those found in pollen. This was unexpected as previous literature suggests a fairly universal blue bias [10, 25, 35]. PR-S bias is important for future behavioral and toxicological free-flight study as it shows that the frequently used blue-white paradigm may not be best suited for all subspecies.

This subspecies analysis provides insight into the toxicological profile of aluminum as well as the differences between two subspecies. Unfortunately, the single dose does not fully predict how bees living in aluminum contaminated regions may respond to exposure. This dose was extremely low (40mg/L) considering the concentrations found in plant products as high as 670mg/kg [33]. The cap pushing paradigm can be used for future toxicological work to understand free-flight patterns, floral manipulation, and color choice. Although aluminum did not affect these metrics at the dose given, it is possible that other contaminants may and this procedure should be considered.

Long-term dosing of bees from contaminated regions is more realistic but cannot be completed in the field without risk of whole hive contamination. The monitor system allows us to increase the concentration and longevity of aluminum exposure although is does remove ecosystem variables that free-flight experiments provide. At 40mg/L OK-M bees were the only subspecies affected as compared to two Mediterranean subspecies. However this dose is relatively small, we only used single-colonies, and there was temporal and environmental variation between colonies that may reduce the validity of the statistics. Single-colony comparisons allow us to control for genetic and toxicological variation within a subspecies, however it does not fully illuminate subspecies toxicity. In addition to only single colonies being represented, the varying conditions between the Mediterranean subspecies and OK-M may have contributed to the differential toxicity seen here. For this reason, both within and between subspecies effects were examined to understand how subspecies respond to aluminum exposure.

*Apis mellifera mellifera* (OK-M) appear to be the most susceptible to aluminum exposure of the subspecies tested. For this reason, we used OK-M to generate a more in-depth toxicity curve of aluminum with the highest average concentrations found in pollen in Brazil [34]. Although higher concentrations have been found in plant material, the direct applicability of a pollen concentration is more ecologically relevant. Pollen concentrations were used to estimate the most drastic effects of aluminum exposure; however the experimental delivery method was water. For these reasons, we cannot make direct comparisons to pollen ingestion. However, the similarities between pollen and nectar concentrations do provide some insight into the toxicology of honey bees in contaminated regions [62]. Although the monitor apparatus uses water as the primary exposure route with concentrations above typical water concentrations, the ingestion rate is likely low compared to nectar exposure and is therefore likely a conservative metric [63]. Aluminum significantly reduced survival at 10.4mg/L, much lower than the concentrations found in floral tissues in North America [33]. We predict that the effect on survival is a result of acetylcholinesterase binding however, enzyme assay experiments will need to be completed to confirm.

Mean activity spiked when low concentrations of aluminum were added to the daily water supply. This is likely the result of inhibition of acetylcholinesterase and resultant over-binding of acetylcholine [32]. At higher doses this overstimulation is averaged out and results in exhaustion and low activity levels in the bees. This implies that even low doses have a lasting effect on activity and may actually have a hormetic effect on bees. Hormesis is when there is a stimulative or possibly adaptive intermediate dose range of some toxicant. We see this in the increased activity level however the possible ecological benefits of the hormetic response on motility are likely muted by the reduction in rhythmicity.

Rhythmicity in pollinators can determine whether a hive is effective at collecting enough resources to sustain it. Plant phenology is typically fairly time-specific and bees must be ready to collect from these food resources while the flowers are blooming [55, 56, 67]. Disruption in their wake-rest patterns may also affect learning and may create long-term deficits. Rhythmicity does return to normalcy at moderate doses and we expect that this is the beginning of a downtrend that can be seen at 268mg/L. We expect that as concentrations continue to increase, rhythmicity will significantly decline from controls. The opposing trends of activity and rhythmicity were not expected as aluminum is not considered a biologically active metal. The possible hormesis shown here deserves more scientific attention to better understand how this toxicant affects organisms. Similar hormetic toxicity has been demonstrated in honey bees when given alcohol and some insecticides [68, 69]. These are considered more biologically active but hormesis from any toxicant deserves further investigation. Continued study with multiple colonies and higher doses in the monitor system would help to better understand aluminum toxicity in bees.

For all experiments, replication was at the individual bee level rather than at the colony-level. Within subspecies, experiments were run assuming the use of a single hive though some contamination from secondary hives is possible. The individual level was chosen to most closely match previous behavioral work [10, 70]. Previous studies suggest that timing differs enough between honey bees to warrant individual and small group data in an effort to limit over-estimating behavioral change [71–73] Literature also suggests that within-hive variation is diverse enough to accommodate environmental change and foraging problems which are integral to the proposed studies [57, 73]. Individual data thus has enough variation to show possible challenges of aluminum exposure without added variability of multiple hive genetics. Inclusion of single-subspecies multiple-hive comparisons can be incorporated in future studies to understand tolerance mechanisms however, in this study the aim was to create a broad picture of subspecies differences. Comparing non-allopatric subspecies can also cause significant environmental and temporal differences between the Mediterranean data and Oklahoma data that may affect the toxicant responses by the bees. For this reason we focused on one subspecies for the development of a dose-response curve.

Further investigation into the mechanism of action of aluminum in honey bees should be completed to verify that acetylcholinesterase activity is decreased by exposure. In addition, Inductively Coupled Atomic Emission Spectrometry (ICP-AES) analysis has shown in contaminated regions of the Netherlands average in-bee concentrations of ~9μg/g dry bee [17]. Future work may include this method in cooperation with the monitor system to investigate in-bee concentrations at high levels of exposure. Until this work can take place, the current evidence suggests that precautions must be taken to reduce acidification through industrial and residential emissions and more closely monitor the practices of Bauxite mining to reduce possible exposure to pollinators.

## Supporting information

S1 Video Honey bee completing cap pushing trial at 8x speed. (WMV)
